# Special Issue “Commemorative Issue Celebrating the 20th Anniversary of the Alzheimer’s Foundation of America: Understanding and Treating Alzheimer’s Disease”

**DOI:** 10.3390/medicina60050712

**Published:** 2024-04-26

**Authors:** Allison B. Reiss, Aaron Pinkhasov

**Affiliations:** Department of Medicine and Biomedical Research Institute, NYU Grossman Long Island School of Medicine, Mineola, NY 11501, USA

Alzheimer’s disease (AD) is the most common form of dementia in older persons. It is a relentless, progressive neurodegenerative disorder, leading to cognitive impairment, deterioration of functional capacity and, ultimately, death [1,2]. The underlying causes of AD remain incompletely understood and, despite the allocation of huge resources towards finding a cure, progress has been slow. Pathologically, the AD brain is characterized by the accumulation of extracellular amyloid plaques and intraneuronal neurofibrillary tangles of phosphorylated tau protein [3]. Mitochondrial abnormalities, neuroinflammation, and synaptic dysfunction are also observed [4]. The economic burden and stress on caregivers and loved ones continues to grow as the population ages [5]. This compelling collection of articles provides a unique update on many practical aspects of navigating the care and treatment of persons with AD with a forward-looking perspective on promising therapeutic approaches. 

In this Special Issue, we showcase several studies addressing the caregivers who take on the responsibilities of tending to the needs of a person with AD. This can take a heavy toll [6,7]. In their article, Sánchez-Alcón et al. present a descriptive correlational cross-sectional study of family caregivers studying dementia grief, which is the feeling of loss experienced by the caregiver prior to the physical death of the person with dementia [8]. Based on self-administered questionnaires, they found that dementia grief intensity was correlated to depressive symptoms and caregiver strain. Cohen and his team used a cross-sectional design in an exploratory analysis of the relationships among caregiver burden, physical frailty, race, and behavioral and psychological symptoms (BPSD) [9]. They found that frailty affected caregiver burden and BPSD functioned as a mediator between various predictor variables and caregiver burden. Hellis and Mukaetova-Ladinska present a review article covering the mental and physical demands placed on informal caregivers, with an emphasis on the need for support from within and outside the network of friends and relatives to mitigate some of the stress, anxiety and depression that can accompany caregiving [10].

Three articles draw attention to key aspects of making the diagnosis of AD. De Levante Raphael discusses the role of the primary care physician in recognizing dementia and the obstacles and difficulties involved. The author points out the need to educate primary care physicians so that they can perform cognitive assessment in older adults, detect impairment, and manage care [11]. Cummings and Kinney undertake a review of the rapidly evolving field of AD biomarkers [12]. They summarize the categories of biomarkers and their role in diagnosis, prediction, prognosis and monitoring of AD. Attention is given to the regulatory process in biomarker development, clinical validation, and the transition from use in clinical trials to application in clinical care. Dastgheib et al. report on their pilot study, applying electrovestibulography to 16 patients with AD, 13 with a mixed pathology of AD-cerebrovascular disease (AD-CVD), and 24 healthy age-matched controls [13]. They incorporated a cutoff Montreal Cognitive Assessment score, and then used their pilot electrovestibulography data to develop a hierarchy diagnostic algorithm to classify subjects as AD, AD-CVD, or control, and tested the robustness of the most informative features of the results against a blind testing dataset. They hope to use this algorithm to bring better accuracy to the challenge of distinguishing AD from AD-CVD.

The treatment of AD is the focus of three papers. Stecker gives a perspective on the broad issues in AD and the potential for implementing a new model encompassing large-scale collaborations and big data to achieve desperately needed innovations in treatment [14]. Angelopoulou et al. consider the value of telemedicine as a tool for bringing care to persons with dementia [15]. They point out the advantages of telemedicine in providing broad access to patient-centered, integrated care, especially for those living in remote areas and those with mobility issues. The convenience, reliability and reasonable costs associated with virtual visits are considered, and the limitations, such as need for digital proficiency and internet connectivity, are outlined. Reiss et al. investigate the current status of developments in AD therapy based on our escalating knowledge of brain biology at the molecular and genetic level [16]. They give a synopsis of novel approaches using small molecules, stem cells, repurposed drugs, deep brain stimulation, and dietary measures. New delivery systems and the shifting of resources away from anti-amyloid therapy brings optimism for future progress toward disease-modifying original therapeutics.

Ding et al. examine the value of a plant-based diet in maintaining cognitive health and mental sharpness [17]. In an organized fashion, they lay out the dietary guidelines and recommendations for nutrients and fiber that have been found to benefit brain health. Consideration is also given to the gut–brain axis and microbiome.

Together, the studies in this Special Issue highlight the importance of caregiving, diagnosis, treatment, and lifestyle in AD. We hope that the reader will find this collection to be a useful reference with tools and information dealing with both pragmatic and theoretical aspects of AD management.

As Guest Editors, we thank the distinguished authors who contributed to this Special Issue. We also extend our gratitude to the superb team at *Medicina* for their care in the handling of each manuscript, and for their support of this project.
